# STDP Allows Close-to-Optimal Spatiotemporal Spike Pattern Detection by Single Coincidence Detector Neurons

**DOI:** 10.1016/j.neuroscience.2017.06.032

**Published:** 2018-10-01

**Authors:** Timothée Masquelier

**Affiliations:** CERCO UMR5549 CNRS – Université Toulouse 3, France

**Keywords:** LTD, Long-Term Depression, LTP, Long-Term Potentiation, LIF, leaky integrate-and-fire, SNR, signal-to-noise ratio, STDP, spike-timing-dependent plasticity, spike-timing-dependent plasticity (STDP), leaky integrate-and-fire neuron, coincidence detection, multi-neuron spike sequence, spatiotemporal spike pattern, unsupervised learning

## Abstract

Repeating spatiotemporal spike patterns exist and carry information. How this information is extracted by downstream neurons is unclear. Here we theoretically investigate to what extent a single cell could detect a given spike pattern and what the optimal parameters to do so are, in particular the membrane time constant τ. Using a leaky integrate-and-fire (LIF) neuron with homogeneous Poisson input, we computed this optimum analytically. We found that a relatively small τ (at most a few tens of ms) is usually optimal, even when the pattern is much longer. This is somewhat counter-intuitive as the resulting detector ignores most of the pattern, due to its fast memory decay. Next, we wondered if spike-timing-dependent plasticity (STDP) could enable a neuron to reach the theoretical optimum. We simulated a LIF equipped with additive STDP, and repeatedly exposed it to a given input spike pattern. As in previous studies, the LIF progressively became selective to the repeating pattern with no supervision, even when the pattern was embedded in Poisson activity. Here we show that, using certain STDP parameters, the resulting pattern detector is optimal. These mechanisms may explain how humans learn repeating sensory sequences. Long sequences could be recognized thanks to coincidence detectors working at a much shorter timescale. This is consistent with the fact that recognition is still possible if a sound sequence is compressed, played backward, or scrambled using 10-ms bins. Coincidence detection is a simple yet powerful mechanism, which could be the main function of neurons in the brain.

## Highlights

• Repeating spike patterns exist and are informative. Can a single cell do the readout?
• We show how a leaky integrate-and-fire (LIF) can do this readout optimally.
• The optimal membrane time constant is short, possibly much shorter than the pattern.
• Spike-timing-dependent plasticity (STDP) can turn a neuron into an optimal detector.
• These results may explain how humans can learn repeating visual or auditory sequences.

## Introduction

Electrophysiologists report the existence of repeating spike sequence involving multiple cells, also called “spatiotemporal spike patterns”, with precision in the millisecond range, both *in vitro* and *in vivo*, lasting from a few tens of ms to several seconds (Tiesinga et al., 2008). In sensory systems, different stimuli evoke different spike patterns (also called “packets”) (Luczak et al., 2015). In other words, the spike patterns contain information about the stimulus. How this information is extracted by downstream neurons is unclear. Can it be done by neurons only one synapse away from the recorded neurons? Or are multiple integration steps needed? Can it be done by simple coincidence detector neurons, or should other temporal features, such as spike ranks, be taken into account? Here we wondered how far we can go with the simplest scenario: the readout is done by simple coincidence detector neurons only one synapse away from the neurons involved in the repeating pattern. We demonstrate that this approach can lead to very robust pattern detectors, provided that the membrane time constants are relatively short, possibly much shorter than the pattern duration.

In addition, it is known that mere repeated exposure to meaningless sensory sequences facilitates their recognition afterward, in the visual (Gold et al., 2014) and auditory modalities (Agus et al., 2010, Andrillon et al., 2015, Viswanathan et al., 2016) (see also contributions in this special issue), even when the subjects were unaware of these repetitions. Thus, an unsupervised learning mechanism must be at work. It could be the so called spike-timing-dependent plasticity (STDP). Indeed, some theoretical studies by us and others have shown that neurons equipped with STDP can become selective to arbitrary repeating spike patterns, even without supervision (Masquelier et al., 2008, Masquelier et al., 2009, Gilson et al., 2011, Humble et al., 2012, Hunzinger et al., 2012, Klampfl and Maass, 2013, Kasabov et al., 2013, Nessler et al., 2013, Krunglevicius, 2015, Yger et al., 2015, Sun et al., 2016). Using numerical simulations, we show here that the resulting detectors can be close to the theoretical optimum.

## Formal description of the problem

**Fig. 1:**
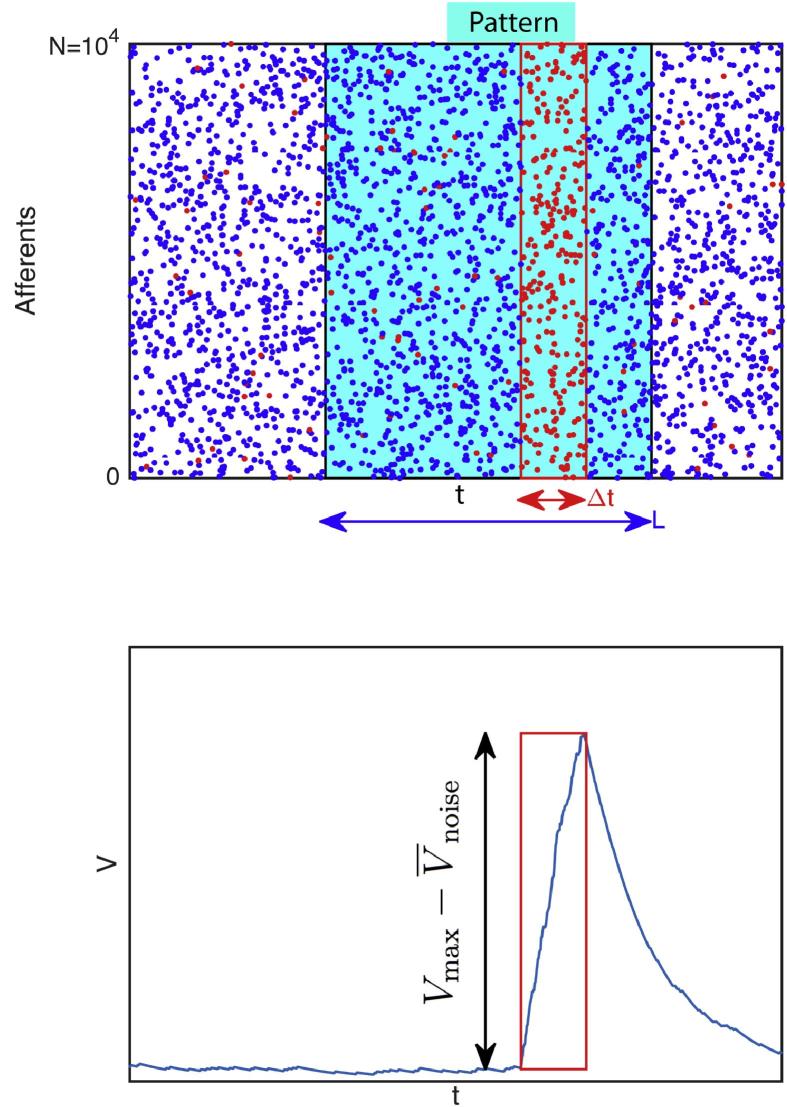
Detecting a spike pattern with a LIF neuron. (Top) Raster plot of $N=10^{4}$ neurons firing according to an homogeneous Poisson process. A pattern of duration *L* can be repeated (frozen noise). Here we illustrated Strategy #1, which consists in connecting the LIF to all neurons that fire at least once during a certain time window of the pattern, with duration Δt⩽L. These neurons emit red spikes. Of course they also fire outside of the Δt window. (Bottom) Typically the LIF’s potential will be particularly high when integrating the spikes of the Δt window, much higher than with random Poisson’s inputs, and we want to optimize this difference, or more precisely the signal-to-noise ratio (SNR, see text).

We assess the problem of detecting a spatiotemporal spike pattern with a single LIF neuron. Intuitively, one should connect the LIF to the neurons that are particularly active during the pattern, or during a subsection of it. That way, the LIF will tend to be more activated by the pattern than by some other input. More formally, we note *L* the pattern duration, *N* the number of neurons it involves. We call Strategy #n the strategy which consists in connecting the LIF to the *M* neurons that emit at least *n* spike(s) during a certain time window Δt⩽L of the pattern. Strategy #1 is illustrated on Fig. 1.

We hypothesize that all afferent neurons fire according to an homogeneous Poisson process with rate *f*, both inside and outside the pattern. That is the pattern corresponds to one realization of the Poisson process, which can be repeated (this is sometimes referred to a “frozen noise”). To model jitter, at each repetition a random time lag is added to each spike, drawn from a uniform distribution over [-T,T] (a normal distribution is more often used, but it would not allow analytical treatment, see next section).

We also assume that synapses are instantaneous (i.e. excitatory postsynaptic currents are made of Diracs), which facilitates the analytic calculations.

For now we ignore the LIF threshold, and we want to optimize its signal-to-noise ratio (SNR), defined as:

(1) $$\mathrm{SNR}=\frac{V_{\max}-{\overset{‾}{V}}_{\text{noise}}}{\sigma_{\text{noise}}}\text{,}$$

where $V_{\max}$ is the maximal potential reached during the pattern presentation, ${\overset{‾}{V}}_{\text{noise}}$ is the mean value for the potential with Poisson input (noise period), and $\sigma_{\text{noise}}$ its standard deviation (see Fig. 1).

## A theoretical optimum

### Deriving the SNR analytically

We now want to calculate the SNR analytically. In this section, we assume unitary synaptic weights. Since the LIF has instantaneous synapses, and the input spikes are generated with a Poisson process, we have ${\overset{‾}{V}}_{\text{noise}}=\tau \mathrm{fM}$ and $\sigma_{\text{noise}}=\sqrt{\tau \mathrm{fM}/2}$, where τ is the membrane’s time constant (Burkitt, 2006). We assume that τfM≫1 (large number of synaptic inputs), so that the distribution of *V* is approximately Gaussian (Burkitt, 2006). Otherwise it would be positively skewed, thus a high SNR as defined by Eq. (1) would not guarantee a low false alarm rate.

The number of selected afferents *M* depends on the strategy *n*. The probability that an afferent fires *k* times in the Δt window is given by the Poisson probability mass function: $\text{P}(k\;\text{spikes})=\frac{\lambda^{k}e^{-\lambda }}{k!}$, with λ=fΔt. The probability that an afferent fires at least *n* times is thus $1-e^{-\lambda }\sum_{k=0}^{n-1}\frac{\lambda^{k}}{k!}$, and finally, on average:

(2) $$M=N\left(1-e^{-\lambda }\overset{n-1}{\underset{k=0}{\sum }}\frac{\lambda^{k}}{k!}\right)\text{.}$$

We now need to estimate $V_{\max}$. Intuitively, during the Δt window, the effective input spike rate, which we call *r*, is typically higher than *fM*, because we deliberately chose the most active afferents. For example, using Strategy #1 with Δt=10ms ensures that this rate is at least 100 Hz per afferent, even if *f* is only a few Hz. More formally, Strategy #n discards the afferents that emit fewer than *n* spikes. This means on average the number of discarded spikes is ${\mathrm{Ne}}^{-\lambda }\sum_{k=0}^{n-1}\frac{k\lambda^{k}}{k!}={\mathrm{Ne}}^{-\lambda }\sum_{k=1}^{n-1}\frac{\lambda^{k}}{(k-1)!}={\mathrm{Ne}}^{-\lambda }\lambda \sum_{k=1}^{n-1}\frac{\lambda^{k-1}}{(k-1)!}={\mathrm{Ne}}^{-\lambda }\lambda \sum_{k=0}^{n-2}\frac{\lambda^{k}}{k!}$. Thus on average:

(3) $$r=N/\Delta t\left(\lambda -e^{-\lambda }\lambda \overset{n-2}{\underset{k=0}{\sum }}\frac{\lambda^{k}}{k!}\right)=\mathrm{Nf}\left(1-e^{-\lambda }\overset{n-2}{\underset{k=0}{\sum }}\frac{\lambda^{k}}{k!}\right)\text{.}$$

We note ${\overset{‾}{V}}^{\infty }=\tau r$ the mean potential of the steady regime that would be reached if Δt was infinite. We now want to compute the transient response. The LIF with instantaneous synapses and unitary synaptic weights obeys the following differential equation:

(4) $$\tau \frac{\text{d}V}{\text{d}t}=-V+\tau \underset{i}{\sum }\delta (t-t_{i})\text{,}$$

where $t_{i}$ are the presynaptic spike times. We first make the approximation of continuity, and replace the sum of Diracs by an equivalent firing rate R(t):

(5) $$\tau \frac{\text{d}V}{\text{d}t}=-V+\tau R(t)\text{.}$$

R(t) should be computed on a time bin which is much smaller than τ, but yet contains many spikes, to avoid discretization effects. In other words, this approximation of continuity is only valid for a large number of spikes in the integration window, that is if rτ≫1, which for Strategy #1 leads to Nfτ≫1.

Note that R(t)=fM during the noise period, and R(t)=r during the Δt window (in the absence of jitter).

At this point it is convenient to introduce the reduced variable $v(t)=\frac{V(t)-{\overset{‾}{V}}_{\text{noise}}}{{\overset{‾}{V}}^{\infty }-{\overset{‾}{V}}_{\text{noise}}}$, which obeys the following differential equation:

(6) $$\tau \frac{\text{d}v}{\text{d}t}=-v+i(t)\text{,}$$

where $i(t)=\frac{R(t)-\mathrm{fM}}{r-\mathrm{fM}}$ is the dimensionless input current, such as i=0 during the noise period (when the input spike rate is *fM*), and i=1 when the input spike rate is *r*).

**Fig. 2:**
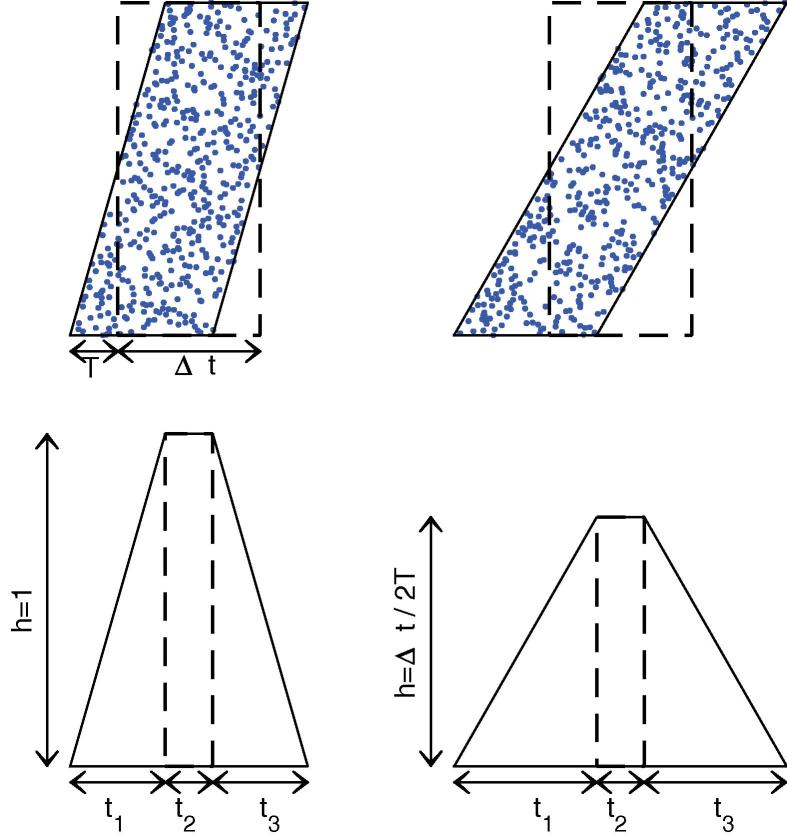
Jittering the spike pattern. (Top) Raster plots for the *M* selected afferents. *x*-Axis is time, and *y*-axis is spike number (arbitrary, so we order them in increasing added jitter, which is a random variable uniformly distributed over [-T,T]). Dashed (resp. solid) lines corresponds to the boundaries of the raster plot before (resp. after) adding jitter. The left (resp. right) panel illustrates the Δt>2T case (resp. Δt<2T). (Bottom) We plotted the corresponding spike time histograms, or, equivalently, doing the approximation of continuity, i(t). One can easily compute $t_{1}=t_{3}=\min(\Delta t\text{,}2T)\text{,}\;t_{2}=|\Delta t-2T|$, and h=min(1,Δt/2T). One can check that the trapezoidal area is Δt whatever *T* (jittering does not add nor remove spikes).

Without jitter, i(t) would be a simple step function, equals to 1 during the Δt window, and 0 elsewhere. Adding jitter, however, turns i(t) into a trapezoidal function, which can be calculated (see Fig. 2). Now that i(t) is known, one can compute v(t) by integrating Eq. (6).

The response of the LIF to an arbitrary current i(t) is (Tuckwell, 1988):

(7) $$v(t)=v_{0}e^{-t/\tau }+1/\tau \int_{0}^{t}e^{-(t-s)/\tau }i(s)\;\text{d}s\text{.}$$

With i=at+b, and given that a primitive of ${\mathrm{te}}^{t}$ is ${\mathrm{te}}^{t}-e^{t}$, the integral can be computed exactly:

(8) $$v(t)=a+b(t-\tau )+(v_{0}-a+b\tau )e^{-t/\tau }\text{.}$$

Note that another jitter distribution than uniform (e.g. normal), would not lead to a piece-wise linear function for i(t), and thus would typically not permit exact integration like here.

**Fig. 3:**
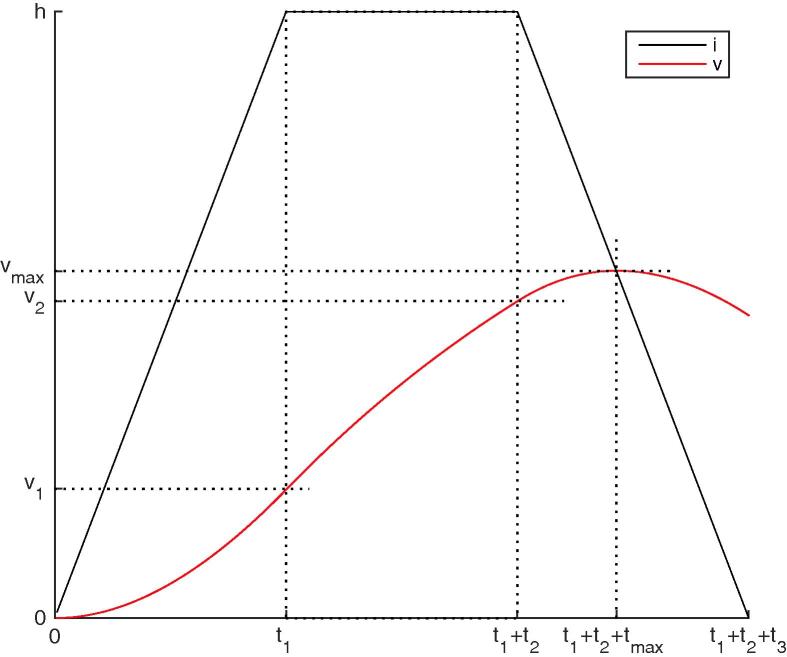
i(t) is piece-wise linear. v(t), which lowpass filters i(t), can be computed exactly on each piece. One can thus compute successively $v_{1}=v(t_{1})\text{,}\;v_{2}=v(t_{1}+t_{2})\text{,}\;v(t)$ for $t_{1}+t_{2}<t<t_{1}+t_{2}+t_{3}$ and its maximum $v_{\max}$, reached for $t=t_{1}+t_{2}+t_{\max}$.

As illustrated on Fig. 3, one can use Eq. (8) to compute successively $v_{1}=v(t_{1})\text{,}\;v_{2}=v(t_{1}+t_{2})$:

(9) $$v_{1}=\frac{t_{1}+\tau (e^{-t_{1}/\tau }-1)}{2T}\text{,}$$

(10) $$v_{2}=h+(v_{1}-h)e^{-t_{2}/\tau }\text{.}$$

One can now compute v(t) for $t_{1}+t_{2}<t<t_{1}+t_{2}+t_{3}$:

(11) $$v(t+t_{1}+t_{2})=h-\frac{t-\tau }{2T}+\left(v_{2}-h-\frac{\tau }{2T}\right)e^{-t/\tau }\text{,}$$

and differentiate it:

(12) $$\frac{\text{d}v(t+t_{1}+t_{2})}{\text{d}t}=-\frac{1}{2T}+\left(\frac{1}{2T}-\frac{v_{2}-h}{\tau }\right)e^{-t/\tau }\text{.}$$

This derivative is 0, indicating that *v* is maximal, for

(13) $$t_{\max}=\tau \log\left(1+2T\frac{h-v_{2}}{\tau }\right)\text{.}$$

One can check that $v_{\max}=h-\frac{t_{\max}}{2T}$ which means that the maximum is on the trapezoid edge, which is logical: before the crossing i>v, so *v* increases; after the crossing i<v, so *v* decreases. Plugging the $t_{\max}$ value into Eq. (11), and expliciting all variables, we have:

(14) $$v_{\max}=\min\left(1\text{,}\frac{\Delta t}{2T}\right)-\frac{\tau }{2T}\log\left(1-e^{-\max(\Delta t\text{,}2T)/\tau }+e^{-|\Delta t-2T|/\tau }\right)\text{.}$$

One can check that if T≪τ and T≪Δt, then $v_{\max}∼1-e^{-\Delta t/\tau }$, which is the classical response of a LIF to a step current.

From the definition of $v:V_{\max}-{\overset{‾}{V}}_{\text{noise}}=v_{\max}(V^{\infty }-{\overset{‾}{V}}_{\text{noise}})$. We now have everything we need to compute the signal-to-noise ratio:

(15) $$\mathrm{SNR}=v_{\max}\frac{V^{\infty }-{\overset{‾}{V}}_{\text{noise}}}{\sigma_{\text{noise}}}=v_{\max}e^{-\lambda }\frac{\lambda^{n-1}}{(n-1)!}\sqrt{\frac{2\tau \mathrm{Nf}}{1-e^{-\lambda }\sum_{k=0}^{n-1}\frac{\lambda^{k}}{k!}}}\text{.}$$

### Numerical validation

**Fig. 4:**
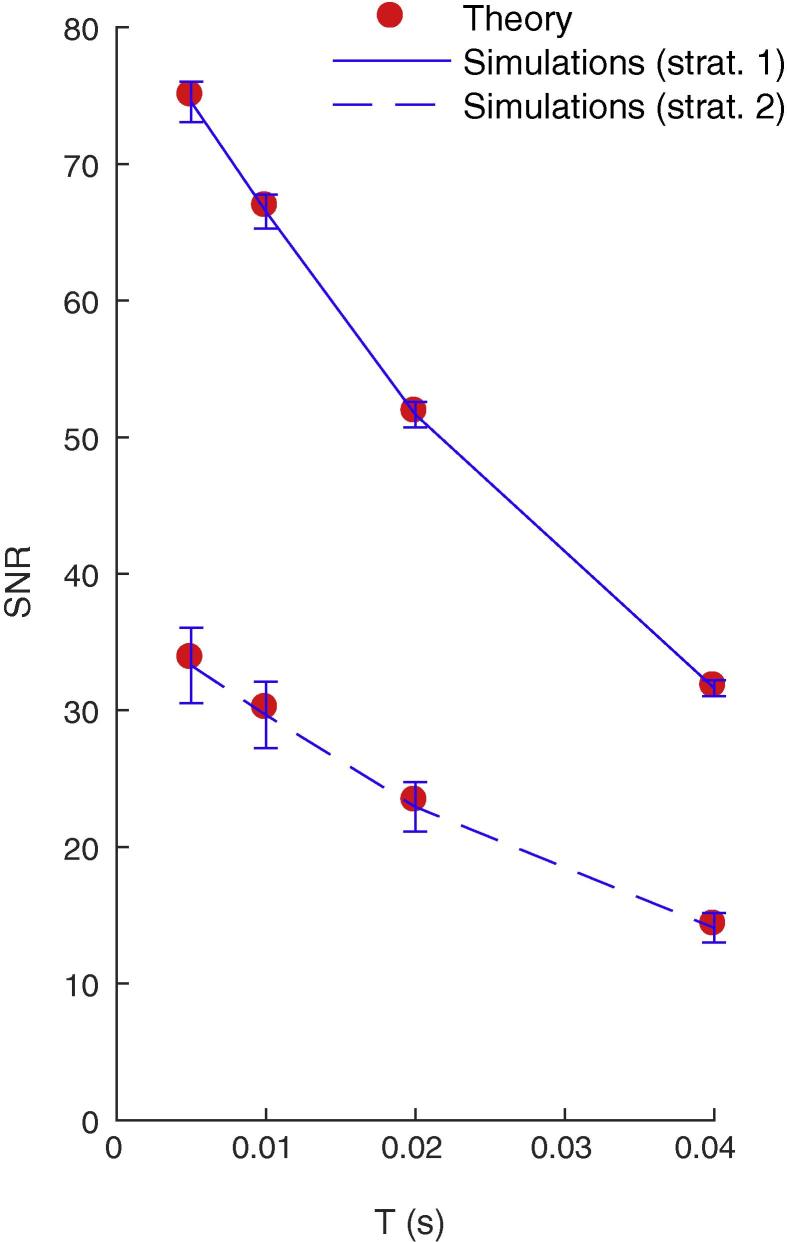
Numerical validation of the theoretical SNR values, for strategies 1 and 2, and for different maximal jitter *T*. Error bars show ±1 s.d.

We verified the exact Eq. (15) through numerical simulations. We used a clock-based approach, and integrated Eq. (4) using the forward Euler method with a 0.1-ms time bin. We generated 100 random Poisson patterns of duration L=20ms, involving $N=10^{4}$ neurons with rate f=5Hz. We chose Δt=L=20ms, i.e. the LIF was connected to all the afferents that emitted at least *n* spikes during the whole pattern, *n* being the strategy number (the constraint τfM≫1 imposes here n⩽2). In order to estimate $V_{\max}$, each pattern was presented 1000 times, every 400 ms. Between pattern presentations, the afferents fired according to a Poisson process, still with rate f=5Hz, which allowed to estimate ${\overset{‾}{V}}_{\text{noise}}$ and $\sigma_{\text{noise}}$. We could thus compute the SNR from Eq. (1) (and its standard deviation across the 100 patterns), which, as can be seen on Fig. 4, matches very well the theoretical values for a broad range of jitters, and for strategies 1 and 2.

### Optimizing the SNR

We now want to optimize the SNR given by Eq. (15). We consider that *f* and *T* are external variables, and that we have the freedom to choose the strategy number n,τ and Δt. We also add the constraint τfM⩾10 (large number of synaptic inputs). We assume that *L* is sufficiently large so that an upper bound for Δt is not needed. We used the Matlab R2015b Optimization Toolbox (MathWorks Inc., Natick, MA, USA) to compute the optimum numerically.

**Fig. 5:**
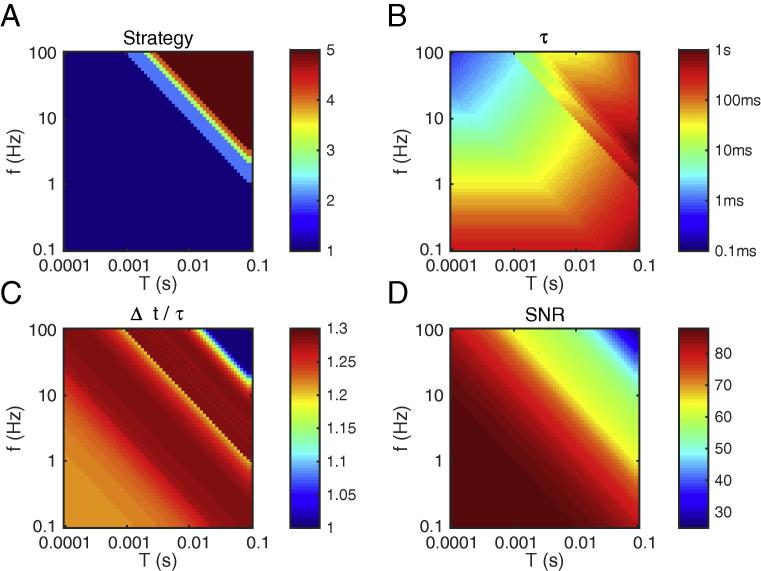
Optimal parameters, as a function of *f* and *T*. (A) Optimal strategy. For clarity we only computed strategies 1…5, but it is clear that higher numbers would be optimal for large *f* and *T*. (B) Optimal τ (note the logarithmic colormap). (C) Optimal Δt, divided by τ. (D) Resulting SNR.

Fig. 5 illustrates the results. One can make the following observations:

• Strategy #1 is usually the best for *f* and *T* in the biological ranges (see below), while higher numbers are optimal for very large *f* and *T* (see panel A). This means that emitting a single spike is already a significant event, that should not be ignored. We will come back to this point in the discussion.
• Unsurprisingly, optimal τ and Δt typically have the same order of magnitude (Δt being slightly larger, see panel C). Unless *T* is high (>10 ms), or *f* is low (<1 Hz), then these timescales should be relatively small (at most a few tens of ms). This means that even a long pattern (hundreds of ms or above) is optimally detected by a coincidence detector working at a shorter timescale. This could explain the apparent paradox between typical ecological stimulus durations (hundreds of ms or above) and the neuronal integration timescales (at most a few tens of ms).
• The constraint τfM⩾10 imposes larger τ when both *f* and *T* are small (panel B, lower left). In the other cases, it is naturally satisfied.
• Unsurprisingly, the optimal SNR decreases with *T*. What is more surprising, is that it also decreases with *f*. In other words, sparse activity is preferable. We will come back to this point in the discussion.

What is the biological range for *f* and *T*? It is worth mentioning that *f* is probably largely overestimated in the electrophysiological literature, because the technique totally ignores the cells that do not fire. Furthermore, experimentalists tend to select the most responsive cells, and search for stimuli that elicit strong responses. *Mean* firing rates, averaged across time and cells, could be smaller than 1 Hz (Shoham et al., 2006). But obviously *some* cells strongly respond to *some* stimuli. The frozen Poisson noise model captures this variability. For example, with L=100ms, f=3.2Hz and $N=10^{4}$ (values used in the next section), leading to λ=0.32 expected spikes per cell, on average the pattern will elicit 0 spike in 7261 cells, 1 spike (10 Hz) in 2324 cells, 2 spikes (20 Hz) in 372 cells, 3 spikes (30 Hz) in 40 cells, and 4 spikes (40 Hz) in 3 cells.

*T* corresponds to the spike time precision. Millisecond precision in cortex has been reported (Kayser et al., 2010, Panzeri and Diamond, 2010, Havenith et al., 2011). We are aware that other studies found poorer precision, but this could be due to uncontrolled variable or the use of inappropriate reference times (Masquelier, 2013).

We now focus, as an example, on the point on the middle of the T×f plane, whose parameters are gathered in Table 1. The resulting SNR is very high (about 80). In other words, it is possible to choose a threshold for the LIF which will be reached when the pattern is presented, but hardly ever in the noise periods.

**Table 1:** Numerical parameters. First two lines correspond to external parameters, the rest of them are parameters to optimize.

Parameter	Value
*T*	3.2 ms
*f*	3.2 Hz

Optimal τ	18 ms
Optimal Δt	23 ms
Optimal *n*	1

In the next section, we investigated, through numerical simulations, if STDP can find this optimum. More specifically, since STDP does not adjust τ, we set it to the optimal value in Table 1 and investigated whether STDP could lead to the optimal *n* and Δt.

## Simulations show that STDP can be close-to-optimal

### Set-up

The set up we used was similar to the one of our previous studies (Masquelier et al., 2008, Gilson et al., 2011). We simulated a LIF neuron connected to all of the $N=10^{4}$ afferents with plastic synaptic weights $w_{i}\in [0\text{,}1]$, obeying the following differential equation:

(16) $$\tau \frac{\text{d}V}{\text{d}t}=-V+\tau \underset{i\text{,}j}{\sum }w_{i}(t_{\mathrm{ij}})\delta (t-t_{\mathrm{ij}})\text{,}$$

Initial synaptic weights were all equal. Then these synaptic weights evolved in [0,1] with additive, all-to-all spike STDP like in Song et al. (2000). Yet we only modeled the Long-Term Potentiation (LTP) part of STDP, ignoring its Long-Term Depression (LTD) term. Here LTD was modeled by a simple homeostatic term $w^{\text{out}}<0$, which is added to each synaptic weight at each postsynaptic spike (Kempter et al., 1999). Note that using a spike-timing-dependent LTD, could also lead to the detection of a repeating pattern, as demonstrated in our earlier studies (Masquelier et al., 2008, Masquelier et al., 2009), but less robustly, because it is more difficult to depress the synapses corresponding to afferents that do not spike in the repeating pattern.

As in Song et al. (2000), at each synapse *i*, we introduce the trace of presynaptic spikes $A_{\text{pre}}^{i}$, which obeys the following differential equation:

(17) $$\tau_{\text{pre}}\frac{\text{d}A_{\text{pre}}^{i}}{\text{d}t}=-A_{\text{pre}}^{i}\text{.}$$

Furthermore:

• At each presynaptic spike: $A_{\text{pre}}^{i}\to A_{\text{pre}}^{i}+\delta A_{\text{pre}}$.
• At each postsynaptic spike: $w^{i}\to w^{i}+A_{\text{pre}}^{i}+w^{\text{out}}$ for i=1,…,N, then the weights are clipped in [0,1].

We used $\delta A_{\text{pre}}=0.01$ and $\tau_{\text{pre}}=20\;\text{ms}$, while $w^{\text{out}}$ and the LIF threshold θ were systematically varied (see below). The refractory period was ignored for simplicity.

We used a clock-based approach, and integrated Eqs. (16), (17) using the forward Euler method with a 0.1-ms time bin. The Matlab code for these simulations has been made available in ModelDB (Hines et al., 2004) at https://senselab.med.yale.edu/modeldb/.

We now describe the way the input spikes were generated. Between pattern presentations, the input spikes were generated randomly with a homogeneous Poisson process with rate *f* (see Table 1). The spike pattern with duration L=100ms was generated only once using the same Poisson process (frozen noise). The pattern presentations occurred every 400 ms (in previous studies, we demonstrated that irregular intervals did not matter (Masquelier et al., 2008, Gilson et al., 2011), so here regular intervals were used for simplicity). At each pattern presentation, all the spike times were shifted independently by some random jitters uniformly distributed over [-T,T] (see Table 1).

### Results: two optimal modes

The theory developed in the previous sections ignored the LIF threshold (a difference of unconstrained potential was maximized). But in the simulations, one needs a threshold to have postsynaptic spikes, necessary for STDP. Since we did not know which threshold values θ could lead to the optimal Δt, we performed an exhaustive search over threshold values, using a geometric progression with a 1.1 ratio. Note that (from Eq. (16)) the threshold θ can be interpreted as the number of synchronous presynaptic spikes needed to reach the threshold from the resting potential if these spikes arrive through maximally reinforced synapses (w=1).

We also used a geometric progression with a 1.1 ratio to search for $w^{\text{out}}$. This parameter tunes the strength of the LTD relative to the LTP, and thus influences the number of reinforced synapses after convergence. For each $\theta \times w^{\text{out}}$ point, 100 simulations were performed with different random patterns, and computed the proportion *p* of “optimal” ones (see below for the definition).

**Fig. 6:**
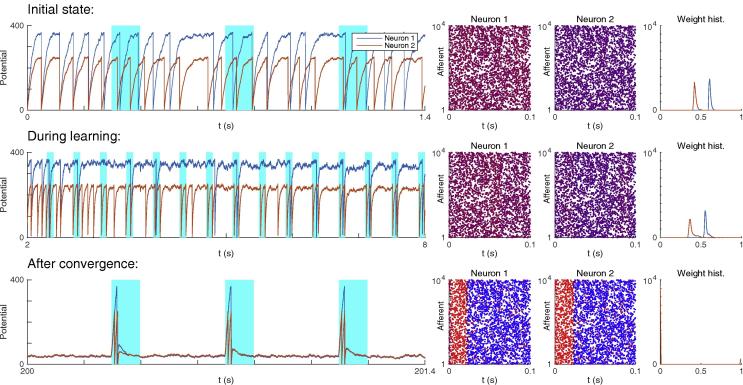
Unsupervised STDP-based pattern learning. Neuron #1 and #2 illustrate modes #1 and #2 respectively. (Top) Initial state. On the left, we plotted the potential of each neuron as a function of time. Cyan rectangles indicate pattern presentations. Next, we represented the weights corresponding to the rightmost time point in two different ways. First, we plotted the spike pattern, coloring the spikes as a function of the corresponding synaptic weight for each neuron: blue for low weight, purple for intermediate weight, and red for high weight. Initial weights were uniform (we used 0.68 for Neuron #1 and 0.47 for Neuron #2, in order to have ${\overset{‾}{V}}_{\text{noise}}=\theta +2\sigma_{\text{noise}}$). We also plotted the weight histogram for each neuron. (Middle) During learning. Selectivity emerges at t∼5s, after ∼12 pattern presentations. Yet the weights still have intermediate values, leading to suboptimal SNR. (Bottom) After convergence. For both neurons, STDP has concentrated the weights on the afferents which fire at least once in a ∼23ms long window, located at the beginning of the pattern. This results in 1 and 2 postsynaptic spikes for Neuron #1 and #2 respectively each time the pattern is presented. Elsewhere both ${\overset{‾}{V}}_{\text{noise}}$ and $\sigma_{\text{noise}}$ are law, resulting in optimal SNR.

The initial weights were computed such that ${\overset{‾}{V}}_{\text{noise}}=\theta +2\sigma_{\text{noise}}$ (leading to an initial firing rate of about 20 Hz, see Fig. 6 top). After 500 pattern presentations, the synaptic weights converged by saturation. That is synapses were either completely depressed (w=0), or maximally reinforced (w=1), as usual with additive STDP (Song et al., 2000, van Rossum et al., 2000, Gütig et al., 2003). A simulation was considered optimal if the reinforced synapses did correspond to a set of afferents which fired at least once (Strategy #1) in a subsection of the pattern, whose duration had to match the optimal Δt window of the pattern given in Table 1 (with a 10% margin). In practice this subsection typically corresponded to the beginning of the pattern, because STDP tracks back through the pattern (Masquelier et al., 2008, Gilson et al., 2011), but this is irrelevant here.

We found two optimal modes (see Fig. 6). The first one, with a high threshold (θ=370) and strong LTD ($w^{\text{out}}=-3.5\times 10^{-3}$) led to 1 postsynaptic spike at each pattern presentation (as in our previous studies (Masquelier et al., 2008, Masquelier et al., 2009, Gilson et al., 2011)). For this mode, p=51%. The second mode, with a lower threshold (θ=250) and weaker LTD $\left(w^{\text{out}}=-1.6\;10^{-3}\right)$ led to 2 postsynaptic spikes at each pattern presentation, and p=87% (the lower threshold increases the probability of false alarms during the noise period, but this problem could be solved by requiring two consecutive spikes for pattern detection). Fig. 6 illustrates an optimal simulation for both modes. We conclude that for most patterns, STDP can turn the LIF neuron into an optimal, or close-to-optimal pattern detector.

Detection is optimal only after convergence (i.e. weight binarization), which takes time (about 500 pattern presentations). This is because the learning rate we used is weak ($\delta A_{\text{pre}}=0.01$, in other words, the maximal weight increase caused by one pair of pre- and post-synaptic spike is only 1% of the maximal weight), as in other theoretical studies and in accordance with experimental measurements (Song et al., 2000, Masquelier et al., 2008, Masquelier et al., 2009, Yger et al., 2015). Using a higher rate, it is possible to converge faster, at the expense of the robustness. For example with $\delta A_{\text{pre}}=0.02$, convergence occurs in ∼250 pattern presentations, but *p* decreases to 44% and 80% for modes #1 and #2 respectively. In any case, it is worth mentioning that (suboptimal) selectivity emerges way before convergence (e.g. around t∼5s, or ∼12 pattern presentations in Fig. 6).

Critically, for successful learning the pattern presentation rate must be high in the early phase of learning, before selectivity emerges. For example presenting the pattern every 800 ms instead of 400 ms leads to p=33% and 43% for modes #1 and #2 respectively. Once selectivity has emerged, this rate has much less impact, since the neuron tends to fire (and thus changes its weights) only at pattern presentations, whatever the intervals between them.

## Discussion

One of the main result of this study is that even a long pattern (hundreds of ms or above) is optimally detected by a coincidence detector working at a shorter timescale (tens of ms), and which thus ignores most of the pattern. One could have thought that using τ∼L, to integrate all the spikes from the pattern would be the best strategy. Instead, it is more optimal to use a subpattern as the signature for the whole pattern (see Fig. 5).

We also demonstrated that STDP can find the optimal signature in an unsupervised manner, by mere pattern repetitions. Note that the problem that STDP solves here is similar to the one addressed by the Tempotron (Gütig and Sompolinsky, 2006), which finds the best spike coincidence to separate two (classes of) patterns, by emitting or not a postsynaptic spike. Recently, the framework has been extended to fire more than one spike per pattern (Gütig, 2016), like here (e.g. Neuron #2 in Fig. 6). Yet these mechanisms require supervision.

In this work we only considered single-cell readout. But of course in the brain, it is likely that a population of cells is involved, and these cells could learn different subpatterns (lateral inhibition could encourage them to do so (Masquelier et al., 2009)). If each cell is selective to a subpart of the repeating pattern, how can one make a full pattern detector? One solution is to use one downstream neuron with appropriate delay lines (Carr and Konishi, 1988). Specifically, the conduction delays should compensate for the differences of latencies, so that the downstream neuron receives the input spikes simultaneously if and only if the sub-patterns are presented in the correct order. Another solution would be to convert the spatiotemporal firing pattern into a spatial one, using neuronal chains with delays as suggested by Tank and Hopfield (1987). Such a spatial pattern – a set of simultaneously active neurons – can then be learned by one downstream neuron equipped with STDP, and fully connected to the neuronal chains, as demonstrated in Larson et al. (2010).

It is also conceivable that the whole pattern is detected based on the mere number of subpattern detectors’ spikes, ignoring their times. Two studies in the human auditory system are consistent with this idea: after learning meaningless white noise sounds, recognition is still possible if the sounds are compressed or played backward (Agus et al., 2010), or chopped into 10-ms bins that are then played in random order (Viswanathan et al., 2016).

Our theoretical study suggests that synchrony is an important part of the neural code (Stanley et al., 2012), that it is computationally efficient (Gütig and Sompolinsky, 2006, Brette, 2012), and that coincidence detection is the main function of neurons (Abeles, 1982, König et al., 1996). In line with this proposal, neurons *in vivo* appear to be mainly fluctuation-driven, not mean-driven (Brette, 2012, Brette, 2015). It remains unclear if other spike time aspects such as ranks (Thorpe and Gautrais, 1998) also matter.

Our results show that, somewhat surprisingly, lower firing rates lead to better signal-to-noise ratio. This could explain why average firing rates are so low in brain, possibly smaller than 1 Hz (Shoham et al., 2006). It seems like neurons only fire when they need to signal an important event, and that every spike matters (Wolfe et al., 2010).
